# Prevalence of Eating Disorders and Their Association with Social Media Addiction among Youths

**DOI:** 10.3390/nu15214687

**Published:** 2023-11-05

**Authors:** Tehreem Mushtaq, Seemab Ashraf, Huma Hameed, Ali Irfan, Maria Shahid, Rabbia Kanwal, Muhammad Arslan Aslam, Hijab Shahid, Koh-E-Noor, Gamal A. Shazly, Mahtab Ahmad Khan, Yousef A. Bin Jardan

**Affiliations:** 1 Faculty of Pharmaceutical Sciences, University of Central Punjab, Lahore 54000, Pakistan; 2 Department of Chemistry, Government College University Faisalabad, Faisalabad 38000, Pakistan; raialiirfan@gmail.com; 3 Department of Pharmaceutics, College of Pharmacy, King Saud University, Riyadh 11451, Saudi Arabia; 4 Institute of Clinical and Experimental Pharmacology and Toxicology, University of Lubeck, 23566 Lubeck, Germany

**Keywords:** eating disorders, social media addiction, anorexia nervosa, bulimia nervosa, social networking site, prevalence

## Abstract

Eating disorders and excessive attachment to social media are a matter of great concern among youths. This study assessed the prevalence of eating disorders and their association with social media addiction among youths. A descriptive cross-sectional study was conducted on 350 participants aged 14–25 years. Two pre-validated tools were used, i.e., the Eating Attitude Test and the Social Networking Addiction Scale. SPSS was used to analyze the data. Out of the 350 students, 42% had probable eating disorders, and 41.7% had social media addictions. The findings revealed that the chances of having eating disorders were significantly higher among youths who lived in separate places, smoked, and had a family history of eating disorders (*p* ≤ 0.05). Furthermore, the dieting domain displayed notably higher scores for youths living separately (*p* ≤ 0.05) and smokers (*p* ≤ 0.01). Moreover, the scores for bulimia and food preoccupation were significantly higher among participants who were married (*p* = 0.038), were smokers (*p* = 0.027), and had a family history of eating disorders (*p* = 0.001). Higher scores in the oral control domain were reported by females (*p* ≤ 0.05) and severely obese youths (*p* ≤ 0.01). Moreover, social media addiction was significantly higher among students aged 18–21 (*p* ≤ 0.01). Spearman’s correlation revealed that social media addiction has a weak positive relationship with eating disorders (*r* = 0.133, *p* ≤ 0.01), particularly bulimia and food preoccupation (*r* = 0.173, *p* ≤ 0.001). This reflects the need to address the harmful consequences of social media addiction that might raise the likelihood of developing eating disorders, particularly bulimia nervosa.

## 1. Introduction

With the advancement of technology, social media platforms have taken center stage in people’s lives. These web-based platforms enable users to build interactive online communities and disseminate all types of information [1]. According to a report in 2023, more than half of the world’s population actively uses social media [2]. In Pakistan, there are almost 72 million active social media users, which is almost 30% of the population [3,4]. Given that youngsters are a part of the digital generation, the most frequent users of social media are also youths. The amount of time that youths spend on social media has increased significantly due to the popularity of these platforms [1,5]. While social media has made it convenient for people to interact and participate in social activities, it has also raised the issue of the excessive usage of social media, posing significant concerns [6,7].

When people become so involved in social media that they are distressed when they cannot use it, it is referred to as social media addiction. The constant usage of these social media platforms promotes addictive behavior among those who use them excessively [8]. This can lead to behavioral and psychological concerns and adversely affect an individual’s mental, social, and emotional well-being [9]. Studies show that, among teenagers who use social media, around 12% exhibit signs of social media addiction [10]. This addiction may affect various aspects of life. One of the alarming concerns about social media addiction is linked to disordered eating patterns among youths. Poor eating behaviors can lead to a greater risk of eating disorders, along with severe psychological and physiological repercussions [11]. Previous research has demonstrated that people who use social media like Facebook are dissatisfied with their appearance and report a higher tendency toward eating disorders [12,13].

Eating disorders can be defined as mental health conditions characterized by severe and irregular food-related practices that are detrimental to one’s physical and mental health. These include anorexia nervosa, bulimia nervosa, and binge-eating disorder [14,15]. Anorexia nervosa is a disorder characterized by an intentionally restricted intake of food and persistent attempts to maintain a body weight less than one’s minimal normal weight or a Body Mass Index (BMI) below 17.5 for one’s age and height. Bulimia nervosa is defined as recurring binge-eating episodes that are followed by recurrent purging, intense exercise, or prolonged fasting at least two times a week for a period of 3 months. Another type of eating disorder is binge-eating disorder, which is defined by frequent binge eating without characteristics like fasting, purging, or rigorous activity [16]. These various eating disorders have the potential to result in numerous mental health issues, such as anxiety, depression, substance abuse, personality disorders, and suicide attempts, as well as various medical complications, including endocrine dysfunction, cardiovascular disease, anemia, and even mortality [16,17]. 

Eating disorders have become a worldwide concern, particularly among young people. The incidence of disordered eating has increased with time, especially among young women. The prevalence of anorexia among female participants in Western countries has been found to range from 0.1% to 5.7%. In Western countries, the prevalence of bulimia nervosa ranged from 0.3% to 7.3% in females, while in males, it ranged from 0.3% to 2.1%. Among non-Western female respondents, the prevalence of bulimia nervosa was around 3.2%. A steady rise is observed in unusual eating habits in non-Western nations [18].

Globally, the younger generation is getting obese, which may also contribute to the growing prevalence of eating disorders in this age group [19]. In addition, Bozkurt et al. stated that young adults who are obese or have a higher BMI have a greater prevalence of internet addiction [20]. Individuals who are addicted to the internet and social media commonly have irregular dietary habits and are used to consuming snacks between meals, leading to obesity [21]. 

A study regarding social media use indicated that men and women spent approximately 3 and 4 h, respectively, using social networking sites (SNSs). Most of that time was spent lurking, which is looking through another user’s profile without actually interacting with them. This study also indicated that the use of social networking sites may be related to body image (BI), self-esteem (SE), and eating disorder (ED) symptoms and concerns [22].

Researchers in France examined the connection of signs of internet addiction with self-esteem, body image avoidance (the wish to avoid situations in which body shape is conspicuous), and disordered eating. Around 10% of men and women were found to have an internet addiction. The symptoms of an internet addiction for both males and females were linked to body image avoidance. Two significant predictors of an eating disorder were social media addiction and body image avoidance [23].

Similarly, a study conducted in Australia reported that 51.7% of girls and 45% of boys had disordered eating behaviors. Moreover, around 75% of girls and 70% of boys owned at least one social media account. Instagram was found to be the most common social media platform. A clear association existed between the usage of social media, DE cognitions, and behaviors at a younger age than previously investigated [24].

The connection between eating disorders among young adolescent boys and girls and social media has not been sufficiently explored in developing countries, especially in Pakistan [25]. This phenomenon is important to explore so that factors leading to eating disorders can be highlighted and awareness can be raised regarding the harmful aspects of excessive social media use [26]. Therefore, the researchers who conducted this study aimed to assess the prevalence of eating disorders and their association with social media addiction among youths in Pakistan. In addition, the roles of demographic factors in different eating disorders and social media addiction were explored.

## 2. Methods

### 2.1. Study Design

A descriptive cross-sectional study was employed to assess the prevalence of eating disorders and their association with social media addiction among youths. 

### 2.2. Study Setting and Respondents

This study was conducted in different schools, colleges, and universities of Pakistan. The participants selected for this study were students aged 14–25 years.

### 2.3. Sample Size and Sampling Technique 

The Raosoft^®^ Sample Size Calculator was used for the calculation of sample size. With a margin of error of 5% and a confidence interval of 95%, the calculated sample size was 382. However, 350 youths took part in the study, and the response rate was 91.6%. The respondents from the study sites were chosen using a non-probability convenience sampling method due to the non-availability of a sampling framework to the researchers. 

### 2.4. Study Tools

A self-administered questionnaire was used comprising a demographic sheet and two pre-validated tools. Demographic data included gender, age, BMI, marital status, socioeconomic status (SES), institution, living place, smoking, and family history of eating disorders. The following pre-validated tools were used.

#### 2.4.1. Eating Attitude Test (EAT-26)

To assess eating disorder risk, a validated tool called the Eating Attitude Test was used. It is a 26-item scale, with each item containing six response options. It has three subscales, i.e., dieting, bulimia and food preoccupation, and oral control. The total score on this scale ranges from 26 to 78. If the score on this tool is 20 or more, or if the BMI is less than the specified criteria with regard to age, it indicates the presence of an eating disorder. The tool was found to be reliable, with Cronbach’s alpha value of α = 0.91 [27].

#### 2.4.2. Social Networking Addiction Scale

The second validated tool utilized for this study was the Social Networking Addiction Scale, comprising 21 items. It is a 7-point Likert scale, with 1 being strongly disagree and 7 being strongly agree. The total score on this scale ranges from 21 to 147. The reliability scores of the scale were found to be α = 0.88. A score greater than 84 indicates social networking addiction [28].

### 2.5. Ethical Considerations 

Research approval for the current study was obtained from the Ethical Committee of the University of Central Punjab (UCP/ORIC/TDF/App#09/2022). Participation in the study was voluntary. Formal informed consent was received from the participants, assuring the anonymity and confidentiality of their responses. 

### 2.6. Data Analysis 

After data collection and entry, the participants’ responses were analyzed using IBM SPSS (Statistical Package for the Social Sciences). Descriptive statistics, including frequency tables, Mann–Whitney U test, Kruskal–Wallis test, and Spearman’s correlation (*p* ≤ 0.05), were applied to analyze the differences or associations between the variables.

## 3. Results

### 3.1. Demographic Characteristics of Participants

Out of 350 participants, 44.9% (*n* = 157) were males, and 55.1% (*n* = 193) were females. Thirty-nine percent of youths (*n* = 137) were aged between 14 and 17 years. Around sixty percent of participants (*n* = 206, 58.9%) had a normal BMI. The majority of the students (*n* = 333, 95.1%) were unmarried. Around ninety percent (*n* = 314, 89.7%) of the participants belonged to the middle class. The data showed that half of the youths (*n* = 178, 50.9%) were studying in universities. Approximately eighty-five percent of the respondents (*n* = 297) were living with their families. Twenty-two out of three hundred and fifty participants (*n* = 22, 6.3%) reported smoking. A small percentage of the participants (*n* = 22, 6.3%) reported having a family history of eating disorders (Table 1).

### 3.2. Assessment of Social Media Use among Youths

The results highlighted that 90.3% (*n* = 316) of the participants use the internet for their education or work. Around fifty percent of the youths (*n* = 161, 46.0%) reported that Instagram was their most preferred social networking site. Almost thirty-eight percent of the participants (*n* = 132) reported using the internet daily for 4–6 h. Lastly, almost half of the youths (*n* = 171, 48.9%) spent 1–3 h daily on social media (Table 2).

### 3.3. Assessment of Eating Disorders and Social Media Addiction among Youths

The findings regarding eating disorders based on the Eating Attitudes Test (EAT-26) highlighted that more than two-fifths of the participants (*n* = 146, 41.7%) had a tendency to develop eating disorders, out of which almost 60% were females, while the remaining 40% were males. A similar percentage of the recruited youth population (*n* = 147) was found to have a social media addiction based on the Social Networking Addiction Scale, in which 85 (57.8%) were females (Table 3).

### 3.4. Comparison of Overall Eating Disorders and Social Media Addiction across Different Demographics

No statistically significant differences (*p* > 0.05) were found in the scores of eating disorders based on gender, age, BMI, marital status, SES, or institution. However, the tendency toward eating disorders significantly differed based on living place (*p* ≤ 0.05). Youths living in separate places had a higher tendency to develop eating disorders. Moreover, the tendency to develop eating disorders was significantly higher in those who smoked (*p* = 0.019). Moreover, those participants who had a family history of eating disorders scored higher on the Eating Attitudes Test, which reflects a higher chance of developing eating disorders (*p* = 0.014). 

No statistically significant differences (*p* > 0.05) were found in social media addiction based on gender, BMI, marital status, social status, institution, living place, smoking, or family history of eating disorders. However, the tendency toward social media addiction significantly differed based on age (*p* ≤ 0.01). Youths aged 18–21 years had higher scores for social media addiction. A detailed description is given in Table 4.

### 3.5. Comparison of Different Domains of Eating Disorders across Different Demographics

No statistically significant differences (*p* > 0.05) were found in the dieting domain based on gender, age, BMI, marital status, SES, institution, or family medical history. However, the tendency toward dieting significantly differed based on living place (*p* ≤ 0.05) and smoking status (*p* ≤ 0.01). Participants or students living in separate places scored higher than those living in hostels or at home. Moreover, students who smoked scored higher in the dieting domain in comparison to non-smokers. 

No statistically significant differences (*p* > 0.05) were found in the bulimia and food preoccupation domain based on gender, age, BMI, socioeconomic status, institution, or living place. However, the tendency toward bulimia and food preoccupation significantly differed based on marital status (*p* ≤ 0.05), smoking status (*p* ≤ 0.05), and family medical history (*p* = 0.001). Youth participants who were married, were smokers, and had a family medical history of eating disorders had a higher probability of developing bulimia. 

No statistically significant differences (*p* > 0.05) were found in the oral control domain based on age, marital status, socioeconomic status, institution, living place, smoking, or family medical history. However, scores in the oral control domain significantly differed based on gender (*p* ≤ 0.05). Females had higher oral control as compared to males. Moreover, the tendency toward oral control significantly differed based on the BMI range (*p* ≤ 0.01). Severely obese youth scored higher in the oral control domain. A detailed description is given in Table 5.

### 3.6. Assessment of the Relationship between Eating Disorders and Social Media Addiction

Social media addiction has a weak positive relationship with overall eating disorders (*r* = 0.133, *p* ≤ 0.01). The association with the specific type of eating disorders revealed that social media addiction has no significant correlation with the dieting and oral control domains. However, a weak positive relationship of social media addiction was found with bulimia and food preoccupation (*r* = 0.173, *p* ≤ 0.001). These results indicate that higher use of social media might lead to eating disorders, especially bulimia and food preoccupation (Table 6).

## 4. Discussion

Eating disorders like anorexia nervosa and bulimia nervosa have emerged as a global concern in the youth population. After substance abuse, eating disorders rank second in the mortality rate of any mental health issue. The current study explored the relationship between social media addiction and eating disorders, along with different contributing factors. It investigated whether social media usage has a relationship with disordered eating behaviors, aiming to generate interventions and promote healthier practices among youths. 

The study included 350 students, with 44.9% (*n* = 157) males and 55.1% (*n* = 193) females. Thirty-nine percent of the respondents were between 14 and 17 years old. Around three-fifths of the participants (*n* = 206) had a normal BMI. The majority were single (95.1%, *n* = 333). Nearly 85% of the people (*n* = 297) in this study resided with their families. Only 6.3% (*n* = 22) of the respondents reported being smokers. Few participants reported having a family history of eating disorders.

The present study revealed that almost half of the youths mostly use Instagram, and 38% of youth use the internet for 4–6 h daily, with a significant amount of time spent on social media. Similar results were found in another study in which Instagram was used by more than 70% of adolescents, and around half of the study population reported daily internet usage of more than 3 h [29]. Youngsters use social media for numerous purposes, i.e., to interact with each other, to communicate and share ideas, to maintain relations with friends, to make new friends, and to acquire new information for academic purposes [30]. One of the significant reasons for the increased usage of social media among youths is the lockdown period during COVID-19. Due to this, the internet and social media became major sources for connecting with friends and relatives and receiving online education from institutions. This could be the reason why youths are now even more engaged in internet usage, mainly social media use [31]. 

The results highlighted that the tendency toward eating disorders is prevalent in 42% of the participants. Similar results were found for the prevalence of social media addiction, indicating that a significant proportion of the recruited youth population has a social media addiction and a tendency to develop eating disorders. Of this 42% of youths, almost 60% were females, while 40% were males. Similar studies conducted in Australia revealed that a significant number of young individuals reported signs of eating disorders, of which 51.7% of girls and 45% of boys had disordered eating behaviors [24]. Another study conducted on social media addiction in India revealed that 32% of the participants had a SNS addiction [32]. Social media addiction is an excessive attachment to activities on social networking sites that can hamper daily functioning. Excessive social media usage can harm users’ biological, physiological, psychological, and social development. Due to this addictive behavior, the lifestyle, perceptions, and habits of youths can be badly affected. The prevalence of eating disorders and social media addiction is significantly increasing, not only in females but in males as well. This vulnerability to becoming addicted to social media and the development of disordered eating could also be due to the possible lack of parental control and monitoring of youths [32,33].

The results of the present study revealed that the prevalence of overall eating disorders significantly varied based on the youth’s living place (*p* ≤ 0.05). People living in separate places had a higher chance of developing eating disorders. With the responsibility of managing their meals, these students may be more susceptible to unhealthy or disordered eating habits. The higher tendency toward eating disorders could also be attributed to factors such as higher levels of stress, academic pressures, and limited control over food choices [34]. 

The results also revealed that the tendency toward eating disorders significantly differed based on smoking status (*p* = 0.019), with smokers scoring higher compared to non-smokers. These findings are consistent with a study conducted in the USA [35]. Smoking may be a compensatory behavior, as appetite is suppressed by nicotine in cigarettes. It also boosts the metabolism and could be a weight control strategy, thus contributing to disordered eating behaviors. There may also be shared psychological factors, such as impulsivity and negative body image, contributing to both smoking and disordered eating behaviors [36]. 

Moreover, the presence of a family history of eating disorders also showed a significant difference (*p* ≤ 0.05), indicating a higher risk. Another study reported similar findings that the chances of developing eating disorders are more than two-fold higher if there is a family history of eating disorders [37]. The presence of a family history may indicate a genetic predisposition, as certain genetic abnormalities and impulsive personality traits have been associated with an increased susceptibility to developing eating disorders [38]. 

The present study also explored different domains of eating disorders. The results revealed significant differences in the dieting domain (categorized by restricted calorie intake, low carbohydrates, and a desire to look slimmer) based on the living place (*p* = 0.012). Youths living in separate places scored higher than those living in hostels or with families. This could be due to a lack of parental guidance and excessive social media use, due to which students could be more impressed by their friends or surroundings and aim to look slimmer in order to be liked and appreciated by others [22]. Moreover, the tendency toward dieting was higher in smokers (*p* ≤ 0.01) as compared to non-smokers. Similar findings were quoted by another study, which highlighted that a greater dependence on nicotine and a higher smoking rate were found in youths with eating disorders in comparison to the control group. This could be attributed to the weight control desire in adolescents [36].

The tendency toward bulimia and food preoccupation (purging after consuming meals and excessive thoughts about food) significantly differed based on family medical history (*p* = 0.001), indicating that students with a family history of eating disorders have a higher likelihood of experiencing bulimia and food preoccupation. These results are consistent with the findings of another study that a person has a 9.6 times higher chance of developing bulimia nervosa if there is a family history of the disorder [39]. 

The results also indicate that the tendency toward bulimia and food preoccupation significantly differed based on marital status (*p* ≤ 0.05) and smoking (*p* ≤ 0.05). Students who were married and were smokers had a higher probability of developing bulimia. Another study conducted in Louisiana, USA, reported that bulimic symptoms and body shape concerns were elevated in smokers. Almost 10% of smokers and 2% of non-smokers reported higher scores on a bulimia-screening test. Furthermore, the percentage of smokers reporting severe body shape concerns was double in comparison to non-smokers. Studies suggest that impaired behavioral regulation, affective instability, impulsivity, and genetic factors may be underlying factors for both smoking and bulimic symptoms. Many females with bulimia nervosa were of the view that smoking is a very useful tool to control weight and appetite [40].

Furthermore, oral control, which means the tendency to self-control eating, significantly differed based on gender (*p* ≤ 0.05), with females scoring higher. Similar results were reflected in another study highlighting that female students, in comparison to males, have a high prevalence of anorexia nervosa, of which oral control is a primary factor [41]. This is because females are less satisfied with their bodies, over-concerned about their weight and shape, and more engaged in behaviors pertaining to body avoidance, due to which they exhibit more oral control tendencies [42]. Moreover, it could also be attributed to sociocultural factors such as societal pressures and unrealistic beauty standards, which affect females more significantly. Through social media platforms, females engage in intensive and regular social comparisons, leading to reduced body satisfaction and disordered eating [23].

The tendency toward oral control also significantly differed based on the BMI range (*p* ≤ 0.01), with severely obese individuals scoring higher in the oral control domain. Deasai et al. also reported that undergraduate students in the USA who were obese were more involved in dieting due to a desire to be thinner in comparison to their peers with normal weight. Disordered eating is generally found to be more prevalent in overweight youths [43]. This emphasizes the importance of considering BMI when examining and addressing eating disorder tendencies among youth. 

The study findings revealed that social media addiction scores were significantly different based on age (*p* ≤ 0.01). Students aged 18–21 scored higher for social media addiction. This is because, in Pakistan, most youths at this age study in universities with large social circles and social gatherings. The internet is easily accessible in universities and at home due to study requirements. Excessive internet use can also lead to the excessive use of social media during free time. Moreover, a lack of parental control at this age can lead to social media addiction to stay up to date regarding the latest trends, influencers, brands, etc.

Lastly, the present study concluded that a weak positive relationship existed between social media addiction and eating disorders, particularly bulimia and food preoccupation (*r* = 0.173, *p* ≤ 0.001). These results suggest that overusing social media platforms may lead to the emergence of disordered eating patterns. Other studies reported similar results [15,22]. A similar study conducted in China showed that both male and female internet addicts reported significantly greater levels of eating disorder symptoms compared to the control groups. These findings suggest that both male and female internet addicts exhibit psychological traits associated with eating problems at considerably higher levels than individuals without an internet addiction [44]. 

Regarding bulimia and food preoccupation, studies have shown that a relationship does exist between SNS usage and bulimia. Due to increased exposure to social media, advertisements, and brand promotions, pressure to reduce weight increases to be more culturally fit. This can precipitate numerous risk factors, like body avoidance, dissatisfaction, dieting, or bulimic symptoms. Social media also greatly affects a person’s self-esteem when people start comparing themselves with other users on social media. This can ultimately lead to low self-esteem and dissatisfaction with life. It is proven by research that low self-esteem is associated with poor eating behaviors and, eventually, eating disorders like bulimia nervosa [45]. In order to reduce the incidence of eating disorders, there is a need to address the possible detrimental effects of social media on people’s mental health and encourage a healthier connection with social media. 

However, there were certain study limitations. The study used self-report measures to collect the data, which might have led to the over- or under-reporting of eating habits and social media use, leading to the possibility of response bias. The researchers opted for a cross-sectional study design due to time constraints and some resource limitations. Lastly, the utilization of clinical assessments of eating disorders might improve the accuracy of the findings. 

## 5. Conclusions

More than 40% of the recruited youth population demonstrated a higher risk of eating disorders and social media addiction, with comparative results in both males and females. Social media addiction was found to have a weak positive relationship with eating disorders, particularly bulimia and food preoccupation. These results suggest that a higher risk of social media addiction may contribute to an increased likelihood of developing eating disorders.

The study also identified several factors associated with eating disorders and social media addiction. Living place, smoking status, and family history of eating disorders significantly influenced the tendency to develop eating disorders. On the other hand, social media addiction was the most prevalent in youths aged 18–21 years. These findings highlight the need to address how social media addiction can be prevented and to raise awareness, educate, and promote healthy online behaviors to mitigate the potential negative consequences of social media addiction, including the development of eating disorders in people, especially youths.

## 6. Research Implications and Recommendations

The assessment of eating disorders and social media addiction holds profound significance in current times. When social media is rigorously used, it can jeopardize young individuals’ mental and physical well-being. This study can raise public awareness among youths regarding the harmful consequences of excessive social media use through targeted advertisements, seminars, and counseling sessions aimed at youths in schools, colleges, and universities. It can serve as a baseline for future investigations, facilitating deeper exploration of the multifaceted factors driving eating disorders via social media. This research gives a reality check to youths regarding eating disorder tendencies, which can help to promote healthier lifestyles among adolescents, safeguard their long-term health, and foster a more balanced relationship with digital media. It is recommended that the authorities monitor social media platforms through content regulation. Collaborating with the advertising industry to promote responsible advertising practices, such as portraying diverse body types, can help reduce body dissatisfaction and eating disorders. It is also essential to increase funding and accessibility to mental health support services, including screening and treatment for eating disorders.

## 7. Future Directions

Future researchers can employ a mixed-methods study approach comprising self-reported questionnaires and in-depth interviews to complement each other and provide a comprehensive understanding of the phenomenon. Longitudinal research can also be conducted to identify the long-term consequences of social media use and how it influences the youth’s thinking patterns and ultimately leads to disrupted eating patterns. Incorporating clinical evaluations and diagnostic criteria would allow for more accurate identification and classification of eating disorder symptoms. 

## Figures and Tables

**Table 1:** Demographic characteristics of participants.

Indicators		*n* (%)
Gender	Male	157 (44.9)
	Female	193 (55.1)
Age in years	14–17	137 (39.1)
	18–21	130 (37.1)
	22–25	83 (23.7)
BMI	Underweight	100 (28.6)
	Normal	206 (58.9)
	Overweight	35 (10)
	Obese	8 (2.3)
	Severely obese	1 (0.3)
Marital status	Single	333 (95.1)
	Married	15 (4.3)
	Divorced	2 (0.6)
Socioeconomic status	Low class	8 (2.3)
	Middle class	314 (89.7)
	High class	28 (8)
Current institution	High school	80 (22.9)
	College	92 (26.3)
	University	178 (50.9)
Living place	With family	297 (84.9)
	Separate place	22 (6.3)
	Hostel	31 (8.9)
Smoking	Yes	22 (6.3)
	No	328 (93.7)
Family history of eating disorders	Yes	22 (6.3)
	No	328 (93.7)

*n* = 350 participants. BMI = Body Mass Index.

**Table 2:** Assessment of social media use among youths.

Indicator		*n* (%)
Do you use the internet for education or work?	Yes	316 (90.3)
	No	34 (9.7)
Social networking site most commonly used	YouTube	146 (41.7)
	Instagram	161 (46.0)
	Twitter	9 (2.6)
	Facebook	34 (9.7)
Daily internet usage time	1–3 h	126 (36.0)
	4–6 h	132 (37.7)
	7–9 h	35 (10.0)
	10–12 h	34 (9.7)
	More than 12 h	23 (6.6)
Daily social media usage time	1–3 h	171 (48.9)
	4–6 h	113 (32.3)
	7–9 h	37 (10.6)
	More than 9 h	29 (8.2)

*n* = 350 participants.

**Table 3:** Assessment of eating disorders and social media addiction among youths.

Indicator	Total *n* (%)	Male *n* (%)	Female *n* (%)
No Eating Disorders	204 (58.3)	98 (48.0)	106 (52.0)
Probable Eating Disorder	146 (41.7)	59 (40.4)	87 (59.6)
No Social Media Addiction	203 (58.0)	95 (46.8)	108 (53.2)
Social Media Addiction	147 (42.0)	62 (42.2)	85 (57.8)

*n* = 350 participants (157 males and 197 females).

**Table 4:** Comparison of overall eating disorders and social media addiction across different demographics.

Indicator	Composite Score	Eating Disorder Total			Social Media Addiction		
	*n*	Mean Rank	Test Statistics	*p* Value	Mean Rank	Test Statistics	*p* Value
Gender ^a^	Male = 157	170.97	14,439.50	0.442	169.15	14,154.00	0.298
	Female = 193	179.18			180.66		
Age in years ^b^	14–17 = 137	163.98	2.92	0.226	155.54	9.45	0.008 **
	18–21 = 130	182.90			192.93		
	22–25 = 83	182.93			181.16		
BMI ^b^	Underweight = 100	177.15	0.47	0.983	192.44	7.69	0.090
	Normal = 206	172.93			164.95		
	Overweight = 35	183.24			175.76		
	Obese = 8	184.31			221.69		
	Severely obese= 1	198.50			276.00		
Marital status ^a^	Single = 333	172.35	1781.50	0.570	175.75	2082.50	0.277
	Married = 15	222.23			146.83		
Socioeconomic status ^b^	Lower class = 8	176.38	5.16	0.070	185.25	0.15	0.930
	Middle class = 314	171.77			175.71		
	High class = 28	217.07			170.30		
Institution ^b^	High school = 80	183.60	1.27	0.527	158.69	4.58	0.101
	College = 92	166.44			169.25		
	University = 178	176.54			186.28		
Living place ^b^	With family = 297	175.52	7.692	0.019 *	177.41	2.22	0.334
	Separate place = 22	221.14			144.43		
	Student hostel = 31	142.97			179.21		
Smoking ^a^	Yes = 22	224.89	2521.50	0.019 *	162.18	3315.00	0.526
	No = 328	172.19			176.39		
Family history of eating disorders ^a^	Yes = 22	226.02	2496.50	0.014 *	183.14	3440.00	0.718
	No = 328	172.11			174.99		

*n* = 350 participants. Mann–Whitney test ^a^, Kruskal–Wallis test ^b^, * *p* ≤ 0.05, ** *p* ≤ 0.01.

**Table 5:** Comparison of eating disorder domains across different demographics.

Indicator	Composite Score	Dieting			Bulimia and Food Preoccupation			Oral Control		
	*n*	Mean Rank	Test Statistics	*p* Value	Mean Rank	Test Statistics	*p* Value	Mean Rank	Test Statistics	*p* Value
Gender ^a^	Male = 157	181.60	14,193.00	0.304	175.16	15,097.00	0.955	162.97	13,183.50	0.035 *
	Female = 193	170.54			175.78			185.69		
Age in years ^b^	14–17 = 137	162.60	4.36	0.112	172.45	0.50	0.784	168.21	1.46	0.485
	18–21 = 130	179.22			174.52			183.10		
	22–25 = 83	190.96			182.07			175.63		
BMI ^b^	Underweight = 100	159.32	7.06	0.113	174.82	1.17	0.918	202.28	14.642	0.004 **
	Normal = 206	177.81			174.68			168.43		
	Overweight = 35	206.70			173.24			149.19		
	Obese = 8	193.06			208.94			121.00		
	Severely obese = 1	85.50			223.00			311.00		
Marital status ^a^	Single = 333	172.73	1906.50	0.115	172.15	1715.00	0.038 *	172.79	1929.50	0.135
	Married = 15	213.90			226.67			212.37		
Socioeconomic status ^b^	Lower class = 8	200.25	2.69	0.260	213.50	5.21	0.074	125.63	5.83	0.054
	Middle class = 314	172.50			171.39			173.49		
	High class = 28	202.02			210.68			212.30		
Current institution ^b^	High school = 80	188.28	3.44	0.175	188.66	1.88	0.388	169.46	0.56	0.752
	College = 92	160.30			169.41			173.62		
	University = 178	177.61			172.73			179.19		
Living place ^b^	With family = 297	174.93	8.81	0.012 *	175.96	2.95	0.235	174.88	1.22	0.549
	Separate place = 22	227.36			200.61			196.57		
	Student hostel= 31	144.13			153.31			166.45		
Smoking ^a^	Yes = 22	231.64	2373.00	0.005 **	220.23	2624.00	0.027 *	185.55	3387.00	0.631
	No = 328	171.73			172.50			174.83		
Family history of eating disorders ^b^	Yes = 22	209.64	2857.00	0.103	242.09	2143.00	0.001 ***	199.64	3077.00	0.253
	No = 328	173.21			171.03			173.88		

*n* = 350 participants. Mann–Whitney test ^a^, Kruskal–Wallis test ^b^. * *p* ≤ 0.05, ** *p* ≤ 0.01, *** *p* ≤ 0.001.

**Table 6:** Spearman’s correlation table showing the relationship between eating disorders and social media addiction.

Indicators	M ± SD	1	2	3	4	5
1. Eating disorder total	13.74 ± 9.10	-				
2. Dieting	6.81 ± 5.82	0.814 ***	-			
3. Bulimia and food preoccupation	2.60 ± 2.71	0.640 ***	0.361 ***	-		
4. Oral control	4.35 ± 3.57	0.659 ***	0.242 ***	0.279 ***	-	
5. Social media addiction	79.84 ± 23.60	0.133 **	0.77	0.173 ***	0.051	-

** *p* ≤ 0.01, *** *p* ≤ 0.001.

## Data Availability

All the data are contained in the manuscript.
